# Supplementary material for: Evidence for rate‐dependent filtering of global extrinsic noise by biochemical reactions in mammalian cells
Source: Mol Syst Biol. 2020 May 14;16(5):e9335. doi: 10.15252/msb.20199335 (PMC7224485; doi:10.15252/msb.20199335)
Supplement: Supplementary file 1 — Expanded View Figures PDF [file MSB-16-e9335-s001.pdf]

Expanded View Figures

Figure EV1. Experimental procedures for characterizing FP maturation kinetics.

A Construct designs and plasmid maps for the assay illustrated in Fig 2A. See Materials and Methods for more details regarding molecular cloning.
B Overall procedures of the assay. Monoclonal or polyclonal cell lines were first plated on glass-bottom 24-well plates, which were then continuously imaged for certain periods on the microscope. The resulting images were processed to obtain single-cell traces, which were then fitted to obtain maturation rates in individual cells. Example fitting results were shown in the fourth panel. The entire procedure was repeated for various FPs.
C Experimental procedures and time-course designs for measuring maturation kinetics (top) or for measuring the scaling ratios for fitting purposes (bottom). See Materials and Methods for more details.
D Control experiment showing that the inducer doxycycline does not affect the production or localization of the constitutive FP (FP1). CHO cells were transiently transfected with plasmids containing the constitutively expressed mTurquoise2 (FP1) and the constitutively expressed nuclear labeling iRFP (iRFP-H2B). Doxycycline was added in the middle of the experiment, and the resulting single-cell trajectories were normalized by mean fluorescence. Error bars indicate ± SD (n = 74 cells).
E Distributions of maturation times for 14 FPs. Data in Fig 2D were plotted as histograms. In order to compare between different FPs, each distribution was normalized by its mean value such that it is centered around one.

 © 2020 The Authors

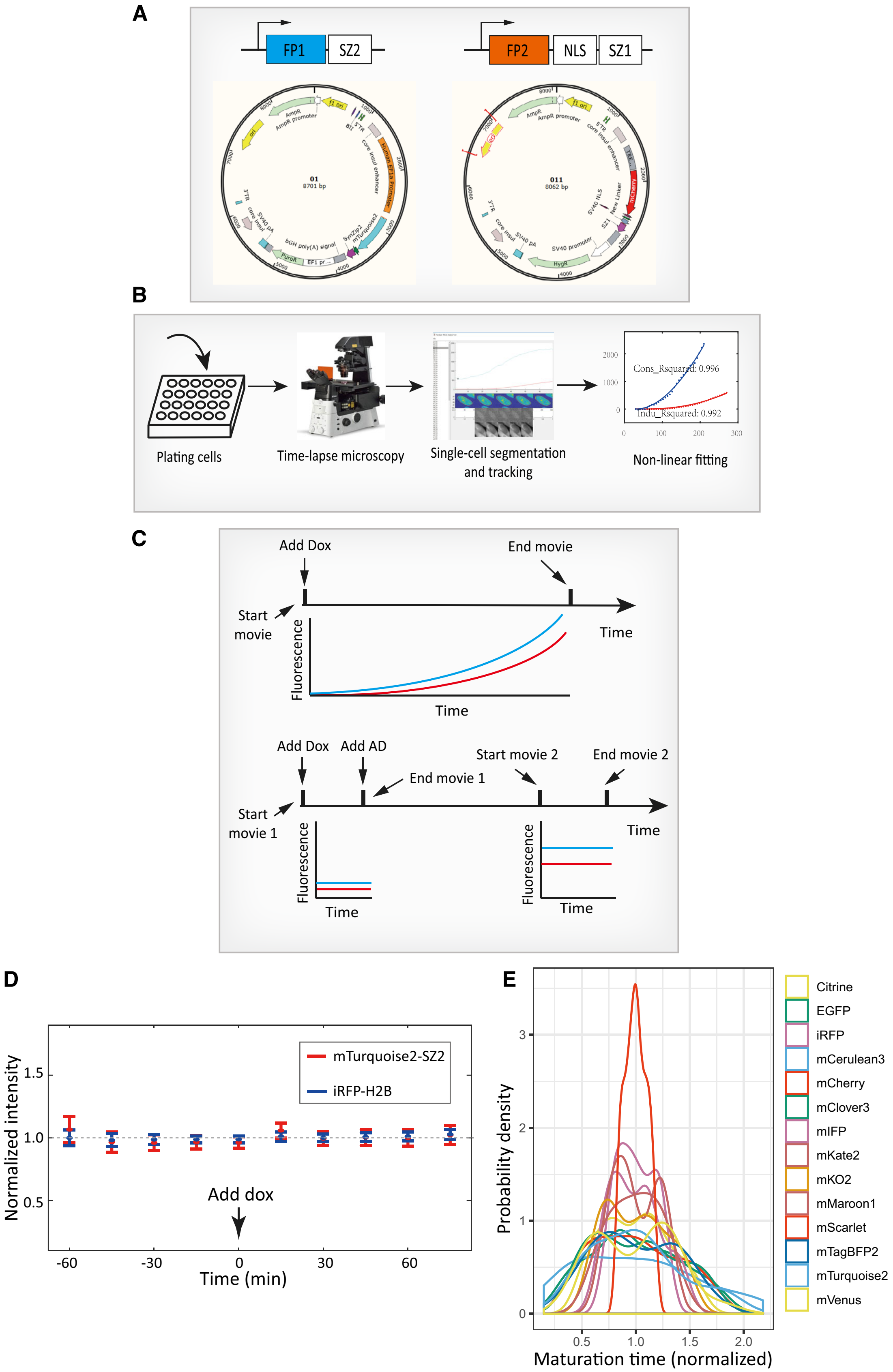
A
FP1
SZ2
FP2
NLS
SZ1
B
Plating cells
Time-lapse microscopy
Single-cell segmentation and tracking
Non-linear fitting
Cons_Rsquared: 0.996
Indu_Rsquared: 0.992
C
Add Dox
End movie
Start movie
Time
Fluorescence
Add AD
Start movie 2
End movie 2
End movie 1
Start movie 1
D
Normalized intensity
mTurquoise2-SZ2
iRFP-H2B
Add dox
Time (min)
E
Probability density
Maturation time (normalized)
Citrine
EGFP
iRFP
mCerulean3
mCherry
mClover3
mIFP
mKate2
mKO2
mMaroon1
mScarlet
mTagBFP2
mTurquoise2
mVenus

Figure EV1.

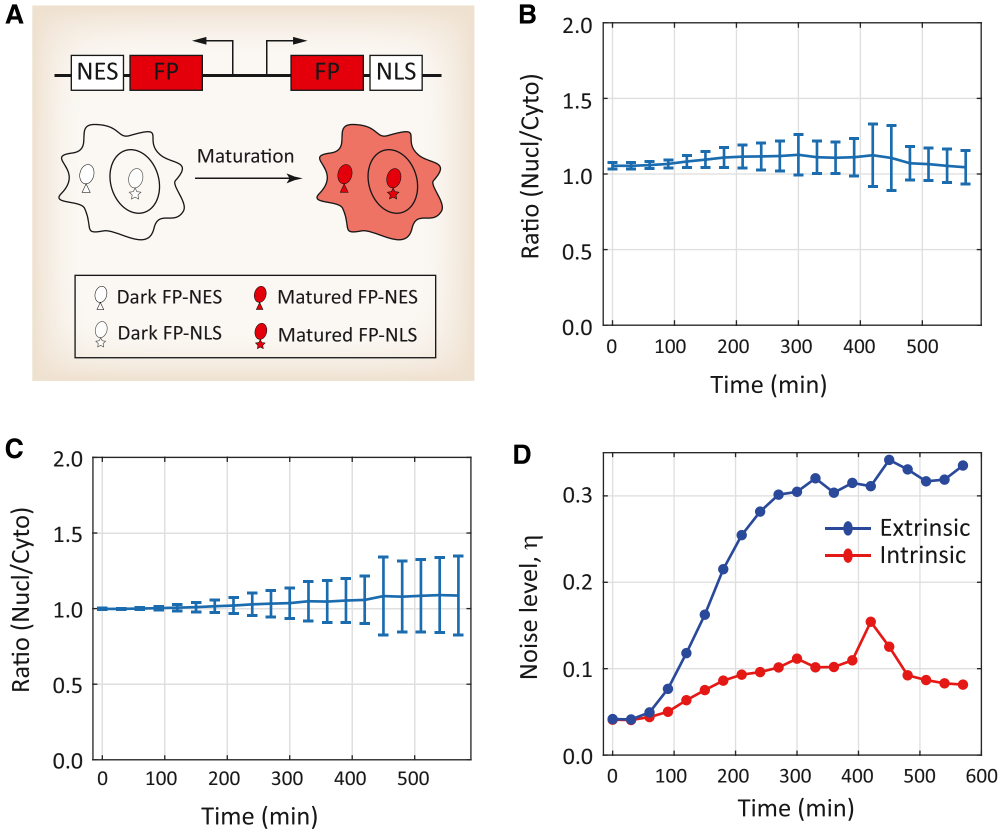

Figure EV2. The maturation rates are similar for both nuclear and cytoplasmic localized FPs.

A Schematic diagram of a bidirectional promoter that drives the expression of two identical FPs but with different subcellular localizations (see Materials and Methods).

B, C Nuclear-to-cytoplasm ratio of mCherry (B) and mTurquoise2 (C) fluorescence signals acquired post-induction at time zero. Error bars indicate ± SD. Datasets in (B) and (C) contain 245 and 135 cells, respectively.

D Analysis of extrinsic and intrinsic noise levels using the dataset in (B). Noise levels (η) were calculated at different time points with the formula described in Elowitz *et al* (2002).

 © 2020 The Authors

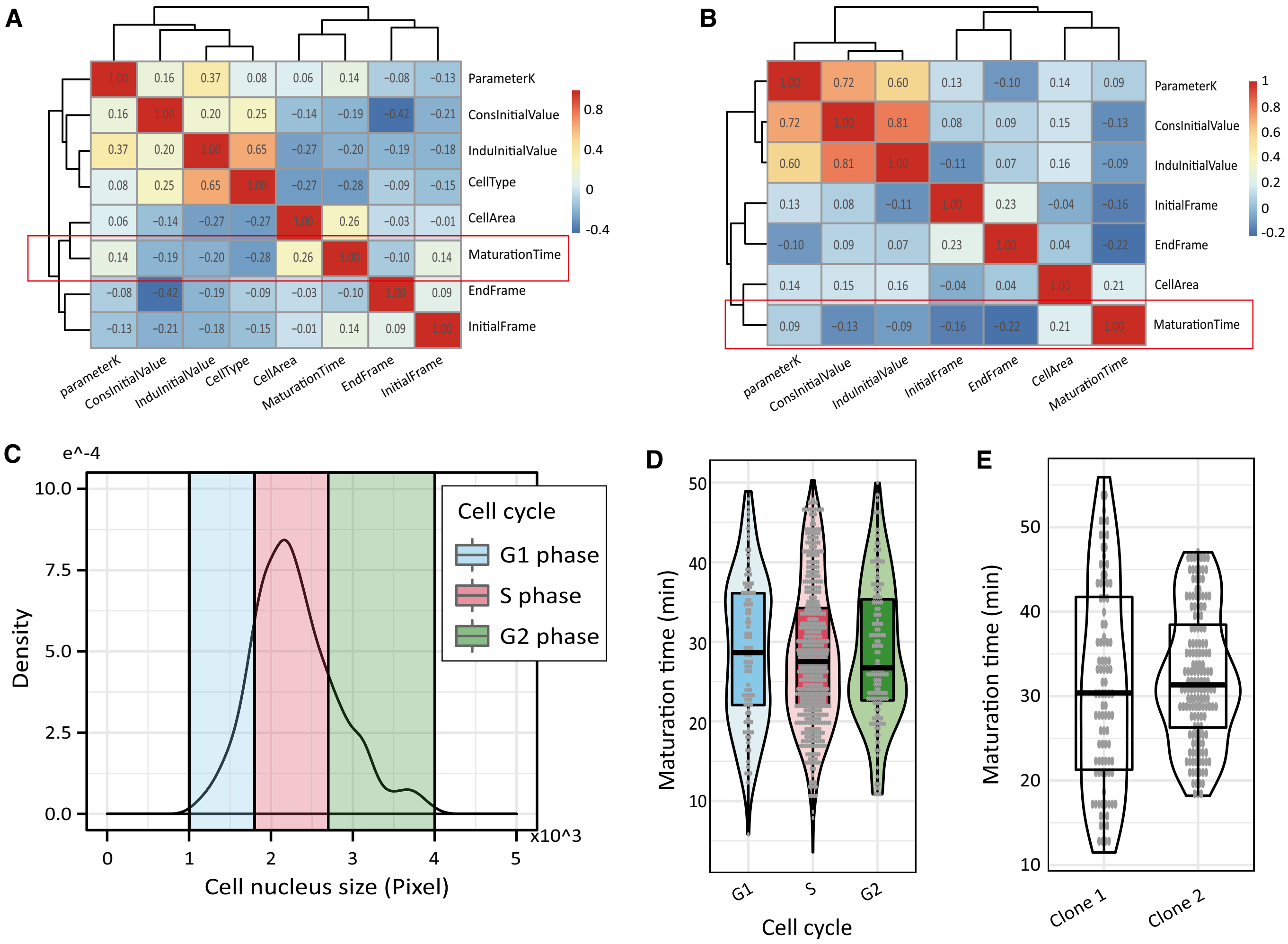

Figure EV3. The maturation kinetics are robust to many cell state-related parameters.

A, B Correlations of single-cell FP maturation time with various cell state-related factors for polyclonal CHO and U2OS cells (A) and for monoclonal CHO cells (B). In both scenarios, the cell state-related parameters showed relatively low correlations with FP maturation kinetics. ParameterK: FP transcription rate multiplied by translation rate; ConsInitialValue: fluorescence value of the constitutively expressed FP in the first time point of fitting; InduInitialValue: fluorescence value of the inducible expressed FP in the first time point of fitting; CellType: different cell types used in our experiment (U2OS and CHO cells); CellArea: cell nuclear size in the first time point of fitting; Maturation Time: measured FP maturation time; EndFrame: the last time point of fitting; InitialFrame: the first time point of fitting. The dataset for (A) and (B) contains 150 and 73 cells, respectively.

C, D Maturation kinetics are not affected by cell cycle stages. (C) Initial nucleus size distribution for cells from cell cycle block-and-release experiments. Cells were then classified into three different cell cycle stages based on initial nucleus size (n = 151, 646, and 201 cells for G1, S, and G2 stage, respectively). (D) mCherry maturation kinetics is robust to cell cycle stage. Cells were classified into different cell cycle stages based on their initial nucleus sizes (n = 151, 646, and 201 cells for G1, S, and G2 stage, respectively). In the violin plot, each box ranges from the first quartile to the third quartile of the data values, and the horizontal line inside indicates the median. The upper whisker is drawn up to the largest data value smaller than the third quartile plus 1.5× the interquartile range (IQR), and the lower whisker is drawn up to the lowest data value larger than the first quartile minus 1.5× IQR. No significant difference was found among different groups (ANOVA test, df = 2, F = 0.179, P = 0.837).

E Comparison of FP maturation kinetics between two different CHO monoclones (n = 101 and 150 two monoclones, respectively). In the violin plot, the definitions of the box and whiskers are the same as in (D). No significant difference for mCherry maturation was found (t = −0.8168, df = 155.72, P = 0.4153).

Figure EV4. Simulation and experimental data supporting rate-dependent filtering of global extrinsic noise.

A Simulated results showing that the non-genetic heterogeneity (coefficient of variation) in FP maturation time increases as the environmental noise level increases.

B Population-averaged fluorescence intensities of the constitutive FP (top) and the inducible FP (bottom) in four different oxyrase treatment conditions (related to Fig 5B). Data from six different time points were shown, and error bars indicate ± SD. Multiple comparison test after Kruskal–Wallis test was performed on these data. NS means no significant difference. * means $P < 0.05$. These results suggest that while FP maturation was affected by oxyrase treatment, FP production was not affected.

C Scatter plot of the noise in FP maturation time and the noise in FP production rate for mCherry in oxygen limitation experiments (Fig 5B, Pearson's correlation coefficient: −0.21, $t = -0.31$, $df = 2$, $P = 0.79$). The dataset for each condition (from right to left) contains $n = 143$, 395, 299, and 529 cells, respectively. All error bars indicate 95% confidence intervals of the mean by bootstrap.

D, E Data from the maturation time measurement of mKate2 in oxygen limitation experiments (analogous to the experiments shown in Fig 5B). The dataset for each condition (from left to right) contains $n = 207$, 238, 193, and 157 cells, respectively. (D) Oxygen level limitation increased the maturation time of mKate2 and decreased the associated heterogeneity. (E) Scatter plot of the noise in FP maturation time and the noise in FP production rate for mKate2 in oxygen limitation experiments (Pearson's correlation coefficient: 0.15, $t = 0.21$, $df = 2$, $P = 0.85$). All error bars indicate 95% confidence intervals of the mean by bootstrap.

© 2020 The Authors

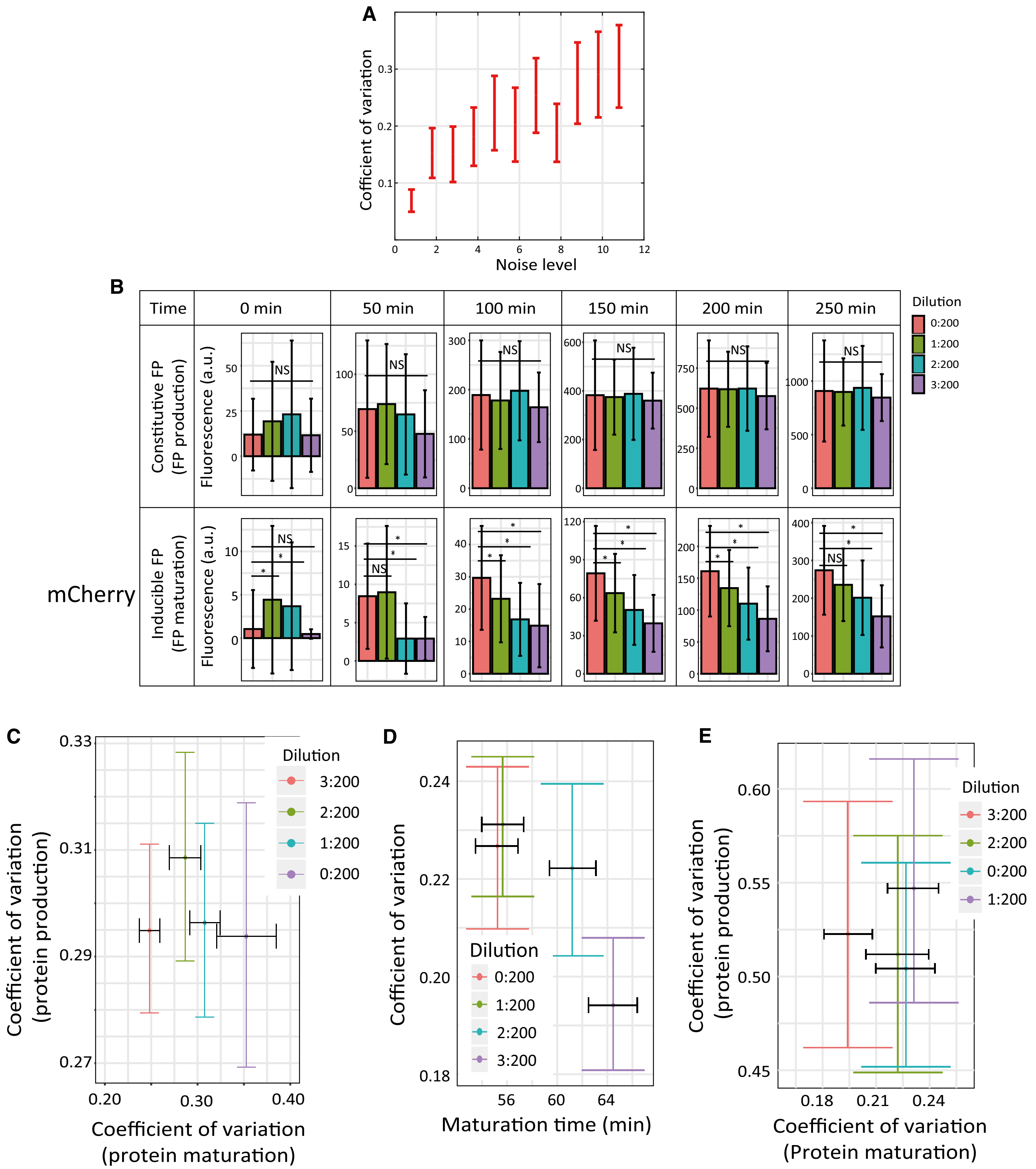
A
Cofficient of variation
Noise level
B
Time
0 min
50 min
100 min
150 min
200 min
250 min
Constitutive FP (FP production)
Inducible FP (FP maturation)
Fluorescence (a.u.)
mCherry
NS
*
Dilution
0:200
1:200
2:200
3:200
C
Coefficient of variation (protein production)
Coefficient of variation (protein maturation)
D
Cofficient of variation
Maturation time (min)
E
Coefficient of variation (protein production)
Coefficient of variation (Protein maturation)

Figure EV4.

Figure EV5. Characterizations of cofactor-dependent maturation of near-infrared FPs.

A, B Population-averaged fluorescence intensities of the constitutive FP (top) and the inducible FP (bottom) under two different cofactor biliverdin concentrations (related to Fig 5D). Data for two separate near-infrared FPs ((A) for iRFP and (B) for mIFP) at six different time points were shown, and error bars indicate ± SD. Multiple comparison test after Kruskal–Wallis test was performed on these data. NS means no significant difference. * means $P < 0.05$. These results suggest that while FP maturation was affected by biliverdin concentration, FP production was not affected.

C Scatter plot of the noise in FP maturation time and the noise in FP production rate for iRFP and mIFP under two different biliverdin conditions (Pearson's correlation coefficient: −0.3542, t = 0.54, df = 2, P = 0.65). The dataset contains n = 116, 39, 56, and 40 cells for mIFP_0, mIFP_10, iRFP_0, and iRFP_10, respectively. Error bars indicate 95% confidence intervals of the mean by bootstrap.

© 2020 The Authors

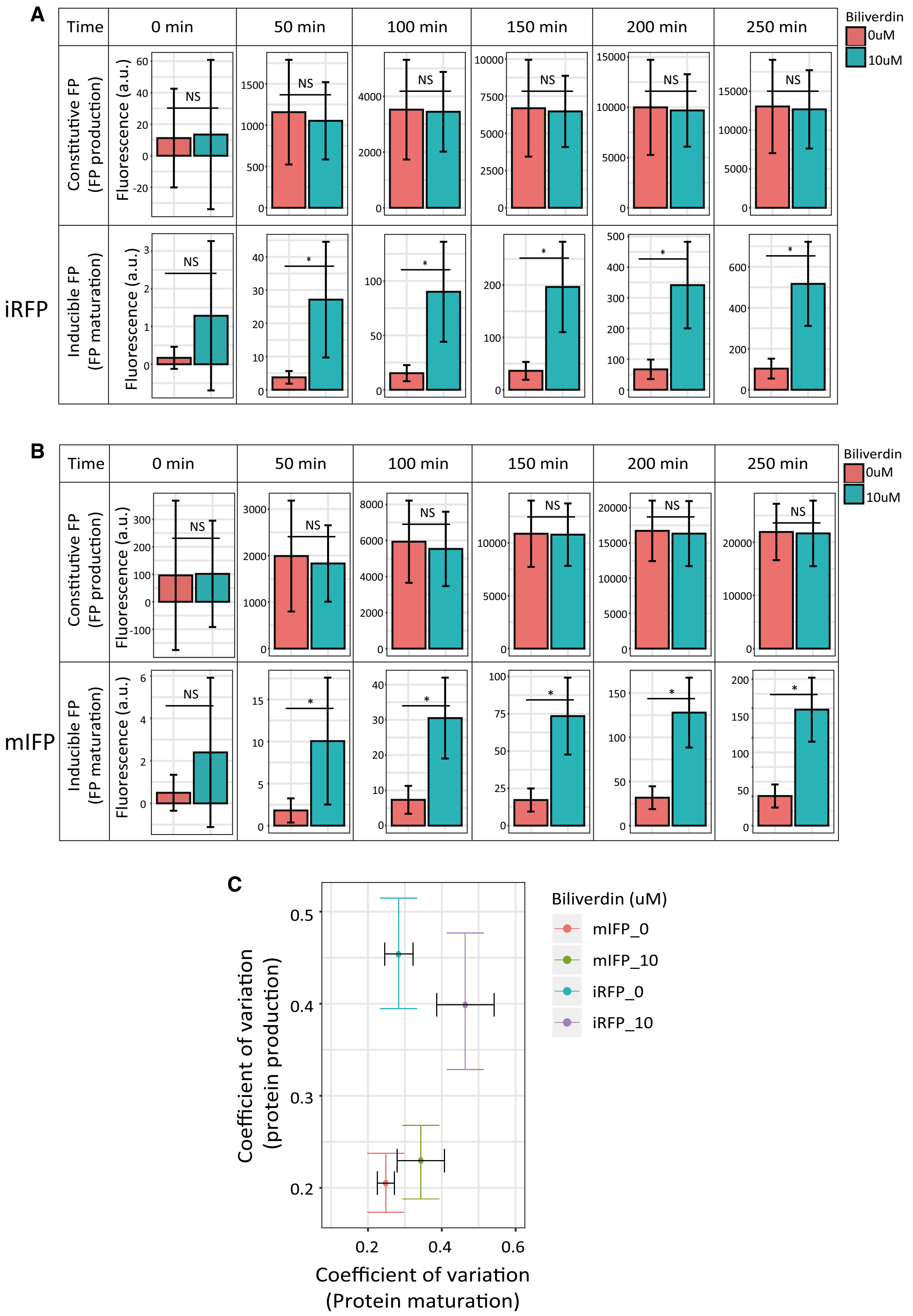
A
Time
0 min
50 min
100 min
150 min
200 min
250 min
Biliverdin
0uM
10uM
Constitutive FP (FP production)
Fluorescence (a.u.)
Inducible FP (FP maturation)
iRFP
NS
*
B
mIFP
C
Coefficient of variation (protein production)
Coefficient of variation (Protein maturation)
Biliverdin (uM)
mIFP_0
mIFP_10
iRFP_0
iRFP_10

Figure EV5.

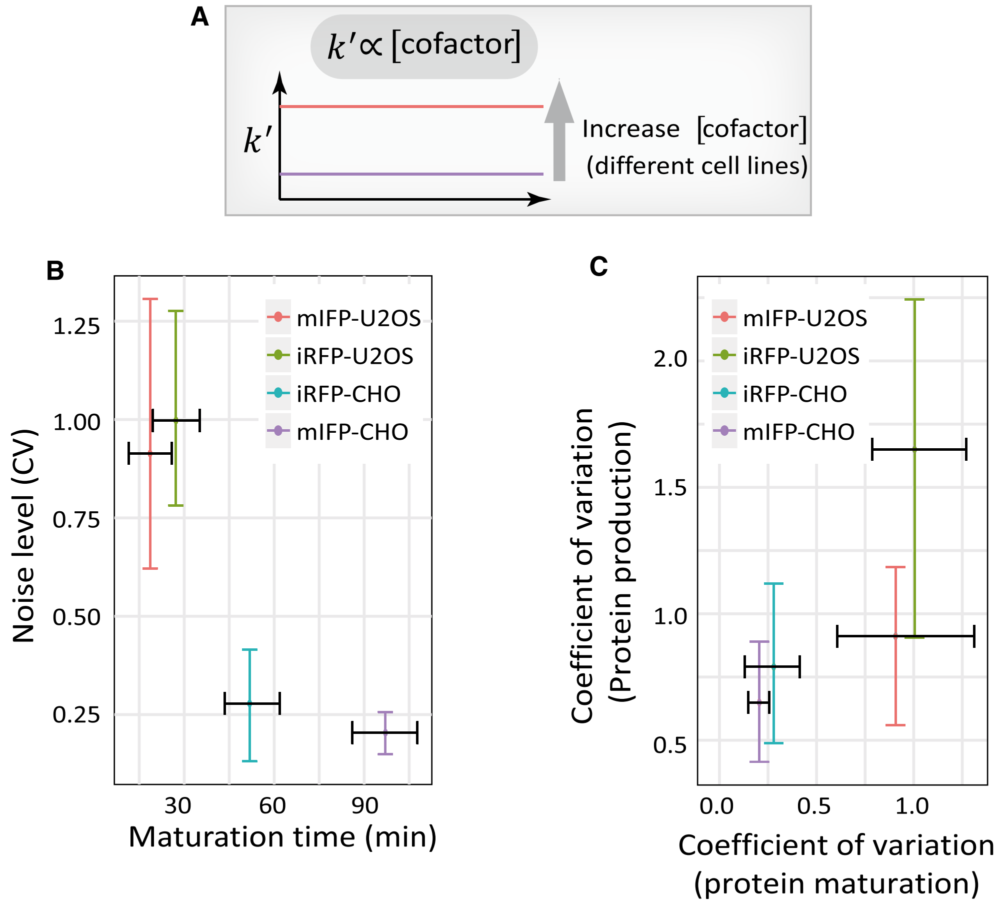

Figure EV6. Characterizations of cell line-dependent maturation of near-infrared FPs.

A The natural variation in the cofactor biliverdin concentration in different cell lines allows us to study the cell line-dependent maturation of near-infrared FPs.

B Cofactor-dependent near-infrared FPs exhibited altered maturation rates and the associated noise levels in a cell line-dependent manner. Both iRFP (t = 3.8101, df = 27.024, P < 0.001) and mIFP (t = 11.576, df = 24.719, P < 0.001) showed significantly different maturation rates in CHO versus U2OS cells. The dataset for each condition (from left to right) contains n = 19, 42, 11, and 14 cells, respectively.

C Scatter plot of the noise in FP maturation time and the noise in FP production rate for two cofactor-dependent FPs (iRFP and mIFP) in two cell lines (CHO and U2OS) (Pearson's correlation coefficient: 0.80 t = 1.88, df = 2, P = 0.20). The dataset for each condition (from left to right) contains n = 14, 11, 19, and 42 cells, respectively.Data information: All error bars indicate 95% confidence intervals of the mean by bootstrap.

© 2020 The Authors
