# Supplementary material for: Evidence for rate‐dependent filtering of global extrinsic noise by biochemical reactions in mammalian cells
Source: Mol Syst Biol. 2020 May 14;16(5):e9335. doi: 10.15252/msb.20199335 (PMC7224485; doi:10.15252/msb.20199335)
Supplement: Supplementary file 2 — Table EV1 [file MSB-16-e9335-s002.zip › Table EV1.pdf]

Table EV 1. Descriptions of cell lines used in our study.

Cell line	Cell type	Transfected FP plasmids	Related figures
d412	CHO (mono-clone)	pTRETightBI-mCherry-3xNLS-mCherry-3xNES;<br>mTurquoise2-3xNLS (Nuclear labeling);	Figure EV2B;<br>Figure EV2D;
d413	CHO (mono-clone)	pTRETightBI-mTurquoise2-3xNLS-mTurquoise2-3xNES;<br>mCherry-3xNLS (Nuclear labeling);	Figure EV2C;
d158	CHO (mono-clone)	PB-pEF1a-mScarlet-SZ2;<br>PB-pTRE3G-mTagBFP2-2xNLS-SZ1;<br>PB-pEF1a-H2B-iRFP;	Figure 2D; Figure 3;
d154	CHO (mono-clone)	PB-pEF1a-mScarlet-SZ2;<br>PB-pTRE3G-mCerulean3-2xNLS-SZ1;<br>PB-pEF1a-H2B-iRFP;	Figure 2D; Figure 3;
d149	CHO (mono-clone)	PB-pEF1a-mScarlet-SZ2;<br>PB-pTRE3G-mTurquoise2-2xNLS-SZ1;<br>PB-pEF1a-H2B-iRFP;	Figure 2D; Figure 3;
d176	CHO (mono-clone)	PB-pEF1a-mScarlet-SZ2;<br>PB-pTRE3G-EGFP-2xNLS-SZ1;<br>PB-pEF1a-H2B-iRFP;	Figure 2D; Figure 3;
d200	CHO (mono-clone)	PB-pEF1a-mScarlet-SZ2;<br>PB-pTRE3G-mClover3-2xNLS-SZ1;<br>PB-pEF1a-H2B-iRFP;	Figure 2D; Figure 3;
d61	CHO (mono-clone)	PB-pEF1a-iRFP-SZ2;<br>PB-pTRE3G-Citrine-2xNLS-SZ1;<br>PB-pEF1a-H2B-mTurquoise2;	Figure 2D; Figure 3;
d94	CHO (mono-clone)	PB-pEF1a-iRFP-SZ2;<br>PB-pTRE3G-mVenus-2xNLS-SZ1;<br>PB-pEF1a-H2B-mTurquoise2;	Figure 2D; Figure 3;
d171	CHO (mono-clone)	PB-pEF1a-mTurquoise2-SZ2;	Figure 2D; Figure 3;

		PB-pTRE3G-mKO2-2xNLS-SZ1;<br>PB-pEF1a-H2B-iRFP;	
d188	CHO (mono-clone)	PB-pEF1a-mTurquoise2-SZ2;<br>PB-pTRE3G-mCherry-2xNLS-SZ1;<br>PB-pEF1a-H2B-iRFP;	Figure 2D; Figure 3;<br>Figure 5B;<br>Figure EV3C-E;<br>Figure EV4B-C;
d69	CHO (mono-clone)	PB-pEF1a-mTurquoise2-SZ2;<br>PB-pTRE3G-mScarlet-2xNLS-SZ1;<br>PB-pEF1a-H2B-iRFP;	Figure 2D; Figure 3;<br>Figure EV4D-E;
d79	CHO (mono-clone)	PB-pEF1a-Citrine-SZ2;<br>PB-pTRE3G-mKate2-2xNLS-SZ1;<br>PB-pEF1a-H2B-mTurquoise2;	Figure 2D; Figure 3;
d59	CHO (mono-clone)	PB-pEF1a-Citrine-SZ2;<br>PB-pTRE3G-mMaroon1-2xNLS-SZ1;<br>PB-pEF1a-H2B-mTurquoise2;	Figure 2D; Figure 3;
d50	CHO (mono-clone)	PB-pEF1a-Citrine-SZ2;<br>PB-pTRE3G-iRFP-2xNLS-SZ1;<br>PB-pEF1a-H2B-mTurquoise2;	Figure 2D; Figure 3;<br>Figure 5D; Figure EV5;
d97	CHO (mono-clone)	PB-pEF1a-Citrine-SZ2;<br>PB-pTRE3G-mIFP-2xNLS-SZ1;<br>PB-pEF1a-H2B-mTurquoise2;	Figure 2D; Figure 3;<br>Figure 5D; Figure EV5;
d192	CHO (mono-clone)	PB-pEF1a-mTurquoise2-SZ2;<br>PB-pTRE3G-mCherry-2xNLS-SZ1;<br>PB-pEF1a-H2B-iRFP;	Figure EV3B;<br>Figure EV3E;
d12	CHO (poly-clone)	PB-pEF1a-Citrine-SZ2;<br>PB-pTRE3G-iRFP-2xNLS-SZ1;<br>PB-pEF1a-H2B-mTurquoise2;	Figure EV6;<br>Figure EV3A;
d13	CHO (poly-clone)	PB-pEF1a-Citrine-SZ2;<br>PB-pTRE3G-mIFP-2xNLS-SZ1;	Figure EV6;

		PB-pEF1a-H2B-mTurquoise2;	
e12	U2OS (poly-clone)	PB-pEF1a-Citrine-SZ2;<br><br>PB-pTRE3G-iRFP-2xNLS-SZ1;<br><br>PB-pEF1a-H2B-mTurquoise2;	Figure EV6;<br><br>Figure EV3A;
e13	U2OS (poly-clone)	PB-pEF1a-Citrine-SZ2;<br><br>PB-pTRE3G-mIFP-2xNLS-SZ1;<br><br>PB-pEF1a-H2B-mTurquoise2;	Figure EV6;
